# Gut Microbiota Profile in Patients with Type 1 Diabetes Based on 16S rRNA Gene Sequencing: A Systematic Review

**DOI:** 10.1155/2020/3936247

**Published:** 2020-08-27

**Authors:** He Zhou, Xue Zhao, Lin Sun, Yujia Liu, You Lv, Xiaokun Gang, Guixia Wang

**Affiliations:** Department of Endocrinology and Metabolism, The First Hospital of Jilin University, Changchun 130021, China

## Abstract

The gut microbiota has been presumed to have a role in the pathogenesis of type 1 diabetes (T1D). Significant changes in the microbial composition of T1D patients have been reported in several case-control studies. This study is aimed at systematically reviewing the existing literature, which has investigated the alterations of the intestinal microbiome in T1D patients compared with healthy controls (HCs) using 16S ribosomal RNA-targeted sequencing. The databases of MEDLINE, EMBASE, Web of Science, and the Cochrane Library were searched until April 2019 for case-control studies comparing the composition of the intestinal microbiome in T1D patients and HCs based on 16S rRNA gene sequencing techniques. The Newcastle-Ottawa Scale was used to assess the methodological quality. Ten articles involving 260 patients with T1D and 276 HCs were included in this systematic review. The quality scores of all included studies were 6–8 points. In summary, a decreased microbiota diversity and a significantly distinct pattern of clustering with regard to *β*-diversity were observed in T1D patients when compared with HCs. At the phylum level, T1D was characterised by a reduced ratio of *Firmicutes/Bacteroidetes* in the structure of the gut community, although no consistent conclusion was reached. At the genus or species level, T1D patients had a reduced abundance of *Clostridium* and *Prevotella* compared with HCs, whereas *Bacteroides* and *Ruminococcus* were found to be more enriched in T1D patients. This systematic review identified that there is a close association between the gut microbiota and development of T1D. Moreover, gut dysbiosis might be involved in the pathogenesis of T1D, although the causative role of gut microbiota remains to be established. Further well-controlled prospective studies are needed to better understand the role of the intestinal microbiome in the pathogenesis of T1D, which may help explore novel microbiota-based strategies to prevent and treat T1D.

## 1. Introduction

Type 1 diabetes (T1D) is a chronic autoimmune disease characterised by the immune-mediated destruction of insulin-producing pancreatic beta cells, usually occurring in children and young adults [1–3]. Although there is still some uncertainty about the aetiology of T1D, it is currently considered a multifactorial autoimmune disorder involving both genetic predisposition and environmental factors [4, 5]. With the introduction of high-throughput sequencing, the structure of microflora can be analysed more comprehensively than before [6]. The intestinal microbiota, known as the “human second genome” [7], can coevolve with their host in a symbiotic relationship by combating pathogenic organisms [8], assisting in food digestion [9], maintaining the integrity of the intestinal epithelia [10], and promoting immunological development [10, 11]. In the past decade, there has been growing evidence suggesting that gut dysbiosis may be a major contributor to T1D development [12]. A variety of studies have identified differences in the gut microbiota of healthy subjects and T1D patients [13]. In addition, growing evidence from well-controlled intervention studies in rodent models has supported the causative association between gut dysbiosis and T1D pathogenesis. The methods commonly used in these studies to alter the composition of gut microbiota include the use of probiotics, the use of antibiotics, fecal microbiota transplantation, and diet intervention. It has been proposed that the altered intestinal microbiota may impact T1D pathogenesis by increasing gut permeability [14], facilitating intestinal inflammation [15], and disturbing immunological maturation [16, 17]. Nevertheless, given the paucity of well-controlled studies in humans owing to the lack of corrective methods for confounding factors, gut microbiota as a causal factor leading to the progression of T1D remains speculative. Once the causative relationship between gut microbiota and T1D development is confirmed and the related pathophysiological mechanisms are delineated, the gut microbiota will be a novel area to explore for new preventative or therapeutic strategies for T1D. The discovery of a clear association between gut dysbiosis and T1D is of significant clinical importance as microbiota-based interventions such as probiotics can reduce or even prevent the burdensome requirement of injected insulin.

The 16S ribosomal RNA (rRNA) gene sequencing technique has become the most widely used method to investigate the composition of microbial ecosystems in recent years [18, 19]. Compared with traditional culture-based methods and previously used low-resolution methods, which can only identify specific bacteria, 16S rRNA-based sequencing as a rapid, cost-effective, and less labour-intensive microbial detection approach can analyse the composition of the whole microbial community and significantly improve the resolution of bacterial identification [6]. To date, a variety of case-control studies have observed perturbed intestinal microflora in T1D patients compared with healthy controls (HCs) using 16S rRNA gene sequencing. Given that different bacterial detection technologies have distinct levels of discrimination, which limits the ability to make accurate and specific comparisons across studies [20], we performed a systematic review to provide an overview of the intestinal microflora profile in patients with T1D based on 16S rRNA gene sequencing.

## 2. Methods

The systematic review was performed according to the PRISMA guidelines (Preferred Reporting Items for Systematic Reviews and Meta-Analyses), and the protocol was registered at PROSPERO (registration number: CRD42019137493).

### 2.1. Literature Search

To identify relevant studies on the intestinal microbial profile associated with T1D, an electronic search was conducted using MEDLINE, EMBASE, Web of Science, and the Cochrane Library databases from inception until April 2019. This search strategy used a combination of MeSH terms and keywords pertaining to gut microbiota and T1D. The following search terms were employed: (“Microbiota” OR “Microbiome” OR “Microflora” OR “Microbes” OR “Microbial Community” OR “Microbiota Composition” OR “Flora” OR “Dysbiosis” OR “Bacteria”) AND (“Type 1 Diabetes” OR “Type 1 Diabetes Mellitus” OR “Autoimmune Diabetes” OR “Insulin-Dependent Diabetes Mellitus” OR “T1DM” OR “Beta-Cell autoimmunity”). The reference lists of the selected studies were manually searched for additional relevant trials.

### 2.2. Eligibility Criteria

The inclusion criteria were as follows: (1) case-control studies comparing gut microbiota in patients with diagnosed T1D and HCs, (2) microbiota analysis using 16S rRNA gene sequencing, and (3) studies published in English.

The exclusion criteria were as follows: (1) studies without HCs; (2) cases being prediabetic subjects with islet autoimmunity, not diagnosed with T1D; (3) microbiota analysis using other microbial detection methods; (4) animal studies; and (5) non-English language studies.

### 2.3. Data Extraction

The following data were extracted from eligible articles by two independent reviewers: (1) first author; (2) year of publication; (3) country of origin; (4) characteristics of cases and HCs (including sample size, mean age, and sex ratio); (5) sample source; (6) DNA extraction method, sequenced region, sequencing platform, analysis platform, and referred database used in these studies; (6) indices of microbial diversity; and (7) major findings of intestinal microbiome in patients with T1D compared to those in HCs. Discrepancies between the two reviewers were resolved by consulting a third reviewer.

### 2.4. Quality Assessment

The Newcastle-Ottawa Scale (NOS) was used to assess the quality of included studies in this systematic review. The NOS is considered an effective way to evaluate the quality of case-control studies, and a total of nine items are included in NOS. The selection criteria contain four items: (1) the adequate case definition, (2) representativeness of the cases, (3) control selection, and (4) control definition. The comparability criteria include comparability of cases and controls according to the design or analysis. The exposure criteria contain three aspects: (1) ascertainment of exposure, (2) the same method of ascertainment for cases and controls, and (3) nonresponse rate.

## 3. Results

### 3.1. Study Selection

**Figure 1:**
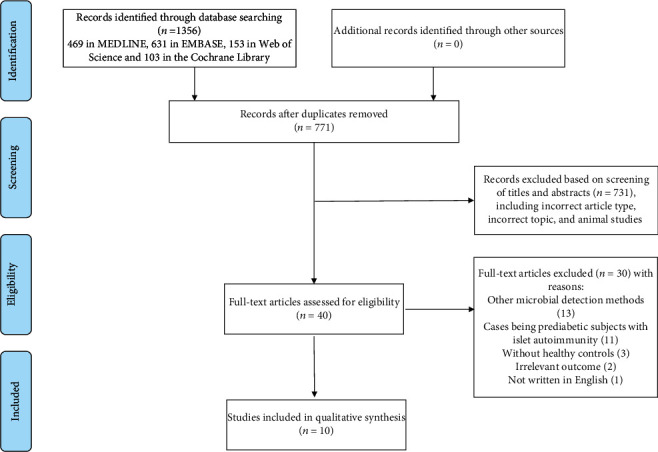
Flowchart of the selected studies.

Following the search strategy, a total of 1356 articles were initially obtained including 469 articles in MEDLINE, 631 in EMBASE, 153 in Web of Science, and 103 in the Cochrane Library. The manual search of reference lists of relevant studies yielded no additional studies. A total of 585 articles were duplicates and were removed. The remaining 771 studies were screened based on the titles and abstracts, of which 731 articles were discarded mainly because of incorrect article types, including editorials, letters, comments, case reports, and reviews as well as incorrect topic and animal studies, leading to 40 articles remaining for full-text review. After reviewing the full text, 30 studies were excluded for the following reasons: (1) 13 studies used other microbial detection methods rather than 16S rRNA gene sequencing; (2) cases of 11 studies were prediabetic subjects with islet autoimmunity, not established T1D patients; (3) 3 studies did not contain a healthy control group; (4) 2 studies provided irrelevant outcomes; and (5) 1 study was not written in English. Finally, a total of 10 studies met the inclusion criteria and were included in this systematic review (Figure 1).

### 3.2. Study Characteristics

The 10 selected articles included a total of 536 individuals with 260 cases of T1D and 276 HCs. The general characteristics and main findings of the included articles are summarised in Table 1. The majority of the eligible studies were published within the last three years. Twelve countries were included in the included studies: Azerbaijan, Jordan, Nigeria, Sudan, Brazil, China, Spain, Poland, the Netherlands, Italy, the UK, and Mexico. The mean age of the patients with T1D ranged from 11 to 36 years and 11 to 38 years in HCs. The proportion of male participants varied from 27.3% to 100% in patients with T1D and from 30.4% to 100% in HCs; additionally, one study was excluded from the gender calculation because its gender description was not available. Except for one study using mucosal biopsies, other studies analysed fecal samples to assess shifts in the gut microbiota composition. The QIAamp DNA Stool Mini Kit was most extensively used for DNA extraction. Different 16S rRNA variable regions were targeted for DNA amplification: region V1–V2 (1 study), region V2–V3 (1 study), region V3–V4 (4 studies), region V3–V5 (1 study), and region V4 (3 studies). Seven studies chose the Illumina MiSeq platform as the sequencing platform, and three studies were performed on the 454 platform. Qiime and Mothur were most widely adopted with respect to data analysis platforms. Although two studies did not report which database was referred, the remaining studies used the Silva database, the Greengenes database, and the Ribosomal Database Project for mapping the sequences. The most frequently utilised estimator for *α*-diversity was the Chao1 index, followed by the Shannon index, although one study did not provide information on which index was employed. The alterations of gut microbiota mainly focused on the dominant phylum and a variety of different specific bacterial genera or subspecies, that is, *Clostridium*, *Bacteroides*, *Prevotella*, *Bifidobacterium*, *Ruminococcus*, and *Streptococcus.* The quality scores of the eligible studies assessed by NOS are shown in Table 2, and all included studies showed medium (6–7 points) to high (8 points) quality.

### 3.3. Microbiota Diversity in Patients with T1D

The richness, evenness, and *α*-diversity can all be used to express the diversity of species. All included studies in this systematic review have investigated the bacterial diversity in T1D patients compared with HCs, although different indexes were utilised. One study conducted by Leiva-Gea et al. found that one of the evidential gut microbiome signatures of patients with T1D was the decreased *α*-diversity estimated by the Shannon index in comparison to HCs [24]. Another study by Qi et al. evaluating the microbial diversity using the Shannon and Chao indices showed that there was a reduced microbiota richness when evaluated using the Chao index in T1D patients compared with that in HCs [29]. A *β*-diversity represents the dissimilarity between the two gut communities. A total of six studies in this review have analysed the *β*-diversity of gut microflora, and four of them consistently identified that the gut microbiome of T1D patients showed a remarkably distinct pattern of clustering when compared with that of HCs [22, 24, 25, 30].

### 3.4. Altered Composition of Gut Microbiota in Patients with T1D

The majority of the studies included in the present systematic review have identified the major taxonomic alterations of microbiota structure, especially at the genus or species level. Only two studies identified that the microbial profile in T1D patients did not differ significantly from that in HCs [25, 28]. This may be explained by differences in dietary habits, geographical environments, and sample sources across studies.

A notable alteration of gut bacterial community at the phyla level was the reduction in the *Firmicutes*/*Bacteroidetes* (F/B) ratio in T1D patients compared with HCs, which has been observed in two studies [23, 24]. Two other studies, whose major taxa explained the altered microbiota profile, were reflected at the genus level or species level; both reported that the F/B ratio was not significantly different between the T1D patients and HCs. However, these two studies found some genus or species belonging to the *Firmicutes*, such as *Lachnospira*, *Dialister*, *Intestinimonas*, and *Caulobacterales* were significantly reduced, while those belonging to *Bacteroidetes*, such as *Bacteroides*, were increased [29, 30]. These results suggested that the alterations of *Firmicutes* and *Bacteroidetes* might be involved in T1D development. Nevertheless, conflicting results have also been published. A study evaluating the microbial composition in duodenal biopsies rather than stool samples found that the ratio of F/B was markedly elevated in T1D patients when compared with HCs [27].

At the genus or species level, six studies consistently demonstrated a significant decline in the abundance of butyrate-producing species primarily within *Clostridium* in patients with T1D when compared with corresponding HCs [21–24, 26, 29]. In these studies, the most commonly detected butyrate-producing species are *Roseburia faecis* (a member of *Clostridium* cluster XIVa) and *Faecalibacterium prausnitzii* (a member of *Clostridium* cluster IV). Moreover, *Intestinimonas*, a newly isolated butyrate-producing bacterium, was also found to be significantly lower in T1D patients in a study performed by Qi et al. [29]. The relative abundance of *Prevotella*, a mucin-degrading bacterium and a marker of elevated mucin synthesis, was also significantly lower in T1D patients than in HCs [27, 30]. In contrast, three studies consistently revealed that *Bacteroides* positively correlated with the development of T1D [24, 26, 30]. Several species belonging to *Bacteroides*, including *Bacteroides vulgatus*, *Bacteroides rodentium*, and *Bacteroides xylanisolvens*, were also significantly higher in T1D patients than in HCs [22]. Similarly, the abundance of *Ruminococcus* also showed an increasing trend in T1D patients [24, 26]. However, inconsistent results were also reported in this systematic review. *Bifidobacterium* was found to be significantly lower in T1D patients than in HCs in two studies [22, 24]; however, it was reported to be higher in another study [26]. Two studies found that *Streptococcus* was more abundant in T1D patients than in HCs [24, 27], whereas another study reported contrasting results [26].

### 3.5. Inflammatory Status along with Gut Dysbiosis in T1D

Eligible studies also determined the T1D-specific inflammatory profile along with the altered composition of gut microbiota in T1D patients. Higuchi et al. demonstrated that the levels of inflammatory interleukin-6 (IL-6) were significantly higher in the plasma of T1D patients than in that of HCs. Additionally, the change in IL-6 plasma concentration correlated with the relative abundance of *Ruminococcaceae* and *Ruminococcus* members [22]. The elevated levels of IL-6 in T1D were reinforced by another study in this review, in which the authors identified increased levels of proinflammatory cytokines including IL-1*β*, IL-6, and TNF-*α* and decreased levels of anti-inflammatory cytokines including IL-10 and IL-13, which significantly correlated with different bacterial groups in T1D patients [24]. They also found a dysbiosis-associated increase in zonulin levels, which indicates an increase in the gut permeability [24]. Pellegrini et al. observed an increased expression of genes specific for T1D inflammation in duodenal mucosa biopsies of T1D patients; it was linked to the altered relative abundance of specific gut microbiota [27]. Furthermore, immunohistochemical analysis of the duodenal mucosa confirmed the inflammatory status with a greater monocyte/macrophage lineage infiltration in the tissues of T1D patients than in those of HCs [27].

## 4. Discussion

Both experimental and observational studies have focused on the alterations of the intestinal microbiome in T1D patients because identifying microbial signatures is a critical step towards providing new insight into the diagnostic or therapeutic strategies for T1D. In the present study, considering that the method of bacterial analysis might be an affecting factor for microbial identification, we explored the shifts of the intestinal microbiome in T1D patients based on 16S rRNA-targeted sequencing to minimise the methodology-based heterogeneity. To our knowledge, our study is the first systematic review exploring the relationships between microbial alterations and the development of T1D based on 16S rRNA-targeted sequencing around the world.

Data from eligible studies concordantly determined a reduced microbiota diversity as well as a significantly distinct pattern of microbiota clustering in T1D patients when compared with HCs. Reduced gut microbial diversity has been previously reported in prediabetic subjects with T1D-associated autoantibodies when compared with autoantibody-negative subjects [31–33]. It has also been detected prior to the appearance of autoantibodies in children at risk for T1D [34], indicating that decreased microbial diversity might be involved in the autoimmune process. In addition, the diversity of microbiota is inversely correlated with several immune-related disorders such as atopic eczema [35], inflammatory bowel disease [36], chronic urticaria [37], and allergic asthma [38]. Microbial diversity is a crucial property of a healthy gastrointestinal ecosystem [39]. According to the hygiene hypothesis, advances in medicine and improved sanitation have changed the microbial environment exposure of humans, characterised by a lack of microbial stimulation and reduced microbial diversity in early childhood, eventually leading to an increase in the incidence of allergy and immune-related disorders [40, 41]. However, the exact mechanism of reduced diversity related to the pathogenesis of T1D is not clear and needs more attention in future studies.

At the phylum level, a decreased F/B ratio in the structure of the gut community in T1D cases was observed because of reduced levels of *Firmicutes* and/or increased levels of *Bacteroidetes.* However, consistent conclusions have not been reached owing to the divergence in the results. A lower F/B ratio is consistent in patients with T1D-associated autoimmunity from cohort studies, in which the stool samples were collected prospectively before the onset of T1D among children genetically at risk for this disorder. It was also reported that the ratio of F/B decreased over time in autoantibody-positive children [31, 32]. A study using polymerase chain reaction- (PCR-) denaturing gradient gel electrophoresis and real-time quantitative PCR (RT-qPCR) to detect the gut microbiota also reported a lower F/B ratio in patients with T1D than in HCs [42]. However, despite these findings, the association of such changes in the F/B ratio with T1D has not yet been elucidated. Future studies should confirm whether the decreased F/B ratio is a microbial signature of the gut community of patients with T1D and elucidate its association with T1D.

At the genus or species level, there were consistent changes in the microbial composition in T1D patients compared with HCs. A reduced abundance of *Clostridium* species, especially clusters IV and XIVa, was found in T1D patients. It was found that these microorganisms perform physiological functions by producing butyrate [43]. Commensal microbes in the intestine can ferment dietary fibres and produce short-chain fatty acids (SCFAs) such as acetate, propionate, and butyrate [44]. Butyrate plays a pivotal role in inducing T regulatory (Treg) cell differentiation in the gut mucosa, which could inhibit the immune response by secreting cytokines such as IL-10 [45, 46]. In addition to its anti-inflammatory activity, butyrate has also been recognised for its ability to enhance gut integrity by increasing tight junction (TJ) production [47] as well as facilitating mucin synthesis [48]. A potential pathway by which intestinal microbes influence the tightness of a TJ seems to be through the rise in zonulin concentration, which is a novel indicator of intestinal permeability [49, 50]. *Faecalibacterium* has been regarded as a next generation probiotic owing to its several health-promoting and anti-inflammatory properties [51]. As noted above, there was a specific inflammatory profile along with the altered composition of gut microbiota in T1D patients [22, 24, 27]. The reduction of butyrate-producing species might contribute to T1D progression by increasing the gut permeability and inducing chronic low-grade inflammation, subsequently eliciting a systemic immune response through a greater exposure to bacterial antigens [49, 52, 53]. *Prevotella*, a mucin-degrading bacterium, was found to be lower in T1D individuals than in HCs. In line with our findings, the numbers of *Prevotella* and *Akkermansia* (another mucin-degrading bacteria) were also reduced in autoantibody-positive subjects compared with matched autoantibody-negative controls [32]. The decline of mucus-degrading microbes may be correlated with the decreased biosynthesis of mucus during T1D development. In our systematic review, the relative abundance of *Bacteroides* consistently showed a higher level in patients with T1D. Similar findings were described in antibody-positive individuals when compared with antibody-negative subjects [32, 33]. *Bacteroides* are acetate- and propionate-producing bacteria [54], and their by-products cannot increase mucin production like butyrate [55]. It has been suggested that these bacteria might contribute to T1D development by thinning the mucus layer, increasing the gut permeability, and leading to chronic inflammation, in which case luminal antigens will escape from the gut and eventually promote islet-directed autoimmune responses [32, 56]. In addition, a recent study identified that gut-derived bacterial lipopolysaccharides from *Bacteroides* may suppress the innate immune signalling and endotoxin tolerance. Thus, this precludes the education and maturation of the immune system in early life [57]. As mentioned earlier, *Ruminococcus* also exhibited elevated levels in T1D patients. It has been established that *Ruminococcus* has proinflammatory effects and is positively correlated with irritable bowel syndrome [58]. However, the role of *Ruminococcus* in T1D development has not been well elucidated. Several studies found a reduced number of *Bifidobacterium* and an increased number of *Streptococcus* in T1D patients, whereas no consistent conclusions have been reached. Members of the genus *Bifidobacterium* are believed to confer positive health benefits to their host, and several *Bifidobacterium* taxa have been commonly used as probiotics [59]. There are several mechanisms that explain the inverse correlation between *Bifidobacterium* and *T1D development*: *Bifidobacterium* can produce lactate, which may be transformed into net butyrate [60], thus having the capacity to protect intestinal permeability [61]. Also, *Bifidobacterium* has been documented to promote the generation and function of Treg cells [62] and their subset type 1 regulatory T (Tr1) cells, which can also produce IL-10 [63], thereby preventing intestinal inflammation. While the influence of *Streptococcus* on the development of T1D remains unclear, it was reported to be increased in patients with multiple sclerosis (MS). It was also speculated that *Streptococcus* was involved in the pathogenesis of MS by inducing the differentiation of Th17 cells [64]. Thus, the inconsistency in the numbers of *Bifidobacterium* and *Streptococcus* in T1D patients needs further exploration and confirmation.

Currently, the pancreatic islet autoantibodies are considered the most reliable biomarkers for T1D in both children and adults [65]. The distinct gut microbiota profile in T1D patients may serve as an alternative biomarker for T1D; moreover, stool sampling is easy to perform and noninvasive. Furthermore, the accumulated data suggest that over time, the trajectory of gut microbiota develops in a different way in children with T1D and in those without T1D before the onset of the disease. Thus, the dysbiosis of the gut microbiota could also be potentially used as a promising biomarker for the early diagnosis of T1D in genetically susceptible children. The intestinal microbiota is also a potential preventative and therapeutic target for T1D. Specific bacterial genera such as *Bifidobacterium* and *Lactobacillus* as well as the metabolites produced by gut microbiota, such as SCFAs, can be used as probiotics and prebiotics, respectively, owing to their favourable impact on the gut environment. Thus far, several animal studies have reported the protective effect of probiotics and prebiotics on T1D [66–68]. Recently, a randomised, placebo-controlled trial in children with T1D demonstrated that the administration of prebiotics for at least one year could increase the relative abundance of *Bifidobacterium* and improve the beta cell function [69]. However, such randomised controlled studies are still limited, and further large-scale clinical trials are warranted.

There are a few limitations to our study. First, a major limitation in all studies is the inherent nature of the cross-sectional design, which cannot provide causal relationships, but only an association between the gut microbiota and T1D development. Second, another limiting factor is the heterogeneity between study populations. The studies included in this review were performed across different countries; thus, the dietary habits might vary considerably. Geographical regions and the diet are major factors that can exert a great impact on the gut community, making it difficult to draw a consistent microbiota profile in T1D patients and even in HCs. Third, the sample type was also a source of heterogeneity. In our systematic review, the majority of the studies used fecal samples, and only one study examined the mucosa biopsy of the intestine. It is recognised that the bacterial composition in feces and biopsy samples may differ in the same individual. Additionally, given that the mucosa-epithelia associated microbiota is likely to have a more intimate interplay with the intestinal epithelium and immune cells, the analysis of microflora using biopsies may be more appropriate [70]. Given the heterogeneity of the study characteristics and results, we conducted a narrative systematic review rather than a meta-analysis.

## 5. Conclusions

This review systematically assessed studies determining the alterations of gut microbiota composition based on 16S rRNA-targeted sequencing in T1D patients compared with that in HCs. In summary, the data from included studies support a relationship between microbiota abnormalities and T1D development. A reduced microbiota diversity and a significantly distinct pattern of clustering with regard to *β*-diversity were observed in T1D patients when compared with those in HCs. At the genus level, T1D was characterised by a reduced F/B ratio in the structure of the gut community, although no consistent conclusion was reached. At the genus or species level, T1D patients consistently showed a reduced abundance of *Clostridium* and *Prevotella* when compared with HCs, whereas *Bacteroides* and *Ruminococcus* were found to be more enriched in T1D patients. However, there is not enough consistency in the alteration of *Bifidobacterium* and *Streptococcus*, which needs to be further confirmed in the future. It seems that the gut microbiota profile in T1D patients was associated with impaired epithelial integrity, low-grade inflammation, and autoimmune response, implying that alterations in these bacterial populations might contribute to the pathogenesis of T1D, although the causative role of dysbiosis remains to be established. Further well-controlled prospective studies are warranted to better understand the role of the gut microbiota in the development of T1D, which may help explore new microbiota-based strategies to prevent and treat T1D.

## Figures and Tables

**Table 1:** Summary of all studies included in this systematic review.

Author Year Country	Number Cases Controls	Mean age Cases Controls	Female/male Cases Controls	Sample type	16S rRNA gene sequencing (including DNA extraction, sequenced region, sequencing platform, analysis platform, referred database)	Indices of microbial diversity	Major findings (compared with control)
Cinek [21] 2018 Azerbaijan Jordan Nigeria Sudan	73 103	11.7 (7.8-13.7) 11.3 (8.1-13.7)	37/33 51/52	Fecal samples	PowerSoil DNA Isolation Kit V4 regions Illumina MiSeq platform Qiime Silva database	Numbers of OTUs Chao1 index ACE index Shannon index Simpson index Fisher index	↑*Escherichia* ↓*Eubacterium* and *Roseburia* ↓*Clostridium* clusters IV or XIVa
Higuchi [22] 2018 Brazil	20 28	23.1 ± 8.6 25.2 ± 9.8	14/6 18/10	Stool samples	PowerSoil DNA Isolation Kit V3-V4 regions Illumina MiSeq platform Mothur RDP	Numbers of OTUs Chao1 index	↓*Bifidobacterium* and *Roseburia* ↑*Bacteroides vulgatus*, *Bacteroides rodentium*, *Prevotella copri*, and *Bacteroides xylanisolvens*
Huang [23] 2018 China	12 10	22.58 ± 2.24 25.10 ± 0.41	7/5 5/5	Fecal samples	E.Z.N.A. Mag-Bind Soil DNA Kit V3-V4 regions Illumina MiSeq platform Qiime RDP	Numbers of OTUs Chao index Shannon index Simpson index	↑*Bacteroidetes/Firmicutes* ratio ↓*Faecalibacterium*
Leiva-Gea [24] 2018 Spain	15 13	12.56 ± 3.59 12.25 ± 2.92	8/7 6/7	Fecal samples	QIAamp DNA Stool Mini Kit V2-V3 regions 454 platform Qiime Greengenes database	Shannon index Chao index	↓Microbiota diversity ↑*Bacteroides*, *Ruminococcus*, *Veillonella*, *Blautia*, and *Streptococcus* ↓*Bifidobacterium*, *Roseburia*, *Faecalibacterium*, and *Lachnospira*
Salamon [25] 2018 Poland	22 23	36 (31-47) 37 (31-48)	16/6 16/7	Fecal samples	Genomic Mini AX Stool Spin V3-V4 regions Illumina MiSeq platform Qiime Greengenes database	NA	The microbial diversity and/or composition of fecal samples in T1D did not differ significantly from those in controls
de Groot [26] 2017 Netherlands	53 50	35 ± 9 36 ± 13	25/28 24/26	Fecal samples	Bead-beating protocol V1-V2 regions Illumina MiSeq platform Qiime, Canoco 5, R-studio Database not specified	Shannon index	↑*Bacteriodales*, *Christensenella*, *Bifidobacterium*, *Ruminococcus*, *Christensenellaceae*, and *Chloroplast* ↓*Subdoligranulum*, *Streptococcus*, *Barnesiella*, *Haemophilus*, *Roseburia*, and *Rhodospirillales*
Pellegrini [27] 2017 Italy	19 16	34 (6-65) 38 (10-56)	9/10 9/7	Biopsy samples	mirVana Kit V3-V5 regions 454 platform Qiime Database not specified	Chao1 index	↓*Proteobacteria* and *Bacteroidetes* ↑*Firmicutes* ↑*Firmicutes*/*Bacteroidetes* ratio ↑*Streptococcus* ↓*Prevotella*
Stewart [28] 2017 UK	10 10	27 ± 2 27 ± 2	0/10 0/10	Fecal samples	PowerLyzer™ PowerSoil® DNA Isolation Kit V4 regions Illumina MiSeq platform Mothur Silva database	Shannon index	No significant difference was reported between the bacterial profiles or the Shannon diversity indices of T1D compared with controls
Qi [29] 2016 China	15 15	11.4 ± 3.0 10.5 ± 1.5	7/8 7/8	Fecal samples	E.Z.N.A.®DNA Kit V3-V4 regions Illumina MiSeq Platform Qiime Silva database	Shannon index Chao index	↓Microbiota richness ↑*Blautia* ↓*Haemophilus*, *Lachnospira*, *Pasteurellales*, *Pasteurellaceae*, *Intestinimonas*, *Micrococcales*, *Dialister*, and *Caulobacterales* ↓*Intestinimonas*
Mejia-Leon [30] 2014 Mexico	21 8	12.3 ± 0.64 11 ± 1.04	NA	Stool samples	QIAamp DNA Stool Mini Kit V4 regions 454 platform Qiime RDP	Chao index	↑*Bacteroides* ↓*Prevotella*, *Megamonas*, and *Acidaminococcus*

**Table 2:** Quality assessment of included studies.

Author	Year	Selection	Comparability	Exposure	Total score
Cinek	2018	4	1	2	7
Higuchi	2018	3	1	2	6
Huang	2018	4	1	2	7
Leiva-Gea	2018	4	2	2	8
Salamon	2018	3	1	2	6
de Groot	2017	3	1	2	6
Pellegrini	2017	4	2	2	8
Stewart	2017	3	1	2	6
Qi	2016	4	2	2	8
Mejia-Leon	2014	3	2	2	7

## Data Availability

The data used to support the findings of this study are available from the corresponding author upon request.
